# Efficient Breast Cancer Diagnosis from Complex Mammographic Images Using Deep Convolutional Neural Network

**DOI:** 10.1155/2023/7717712

**Published:** 2023-03-02

**Authors:** Hameedur Rahman, Tanvir Fatima Naik Bukht, Rozilawati Ahmad, Ahmad Almadhor, Abdul Rehman Javed

**Affiliations:** ^1^Department of Computer Games Development, Faculty of Computing and AI, Air University, E9, Islamabad, Pakistan; ^2^Department of Computer Science, Faculty of Computing and AI, Air University, E9, Islamabad, Pakistan; ^3^Diagnostic Imaging and Radiotherapy Program, Therapeutic and Investigation Studies, Faculty of Health Sciences, University Kebangsaan Malaysia, Kuala Lumpur, Malaysia; ^4^Department of Computer Engineering and Networks, College of Computer and Information Sciences, Jouf University, Sakaka 72388, Saudi Arabia; ^5^Department of Cyber Security, Air University, Islamabad, Pakistan; ^6^Department of Electrical and Computer Engineering, Lebanese American University, Byblos, Lebanon

## Abstract

Medical image analysis places a significant focus on breast cancer, which poses a significant threat to women's health and contributes to many fatalities. An early and precise diagnosis of breast cancer through digital mammograms can significantly improve the accuracy of disease detection. Computer-aided diagnosis (CAD) systems must analyze the medical imagery and perform detection, segmentation, and classification processes to assist radiologists with accurately detecting breast lesions. However, early-stage mammography cancer detection is certainly difficult. The deep convolutional neural network has demonstrated exceptional results and is considered a highly effective tool in the field. This study proposes a computational framework for diagnosing breast cancer using a ResNet-50 convolutional neural network to classify mammogram images. To train and classify the INbreast dataset into benign or malignant categories, the framework utilizes transfer learning from the pretrained ResNet-50 CNN on ImageNet. The results revealed that the proposed framework achieved an outstanding classification accuracy of 93%, surpassing other models trained on the same dataset. This novel approach facilitates early diagnosis and classification of malignant and benign breast cancer, potentially saving lives and resources. These outcomes highlight that deep convolutional neural network algorithms can be trained to achieve highly accurate results in various mammograms, along with the capacity to enhance medical tools by reducing the error rate in screening mammograms.

## 1. Introduction

The predominant cause of cancer-related deaths among women globally is breast cancer [1, 2]. According to a report by the World Health Organization's (WHO) cancer research institute, the International Agency for Research on Cancer, in 2018 globally, 17.1 million breast cancer cases were reported. The number of cases is predicted to increase to double the amount by 2025 [3]. Breast cancer is a highly invasive tumor that primarily affects women [4]. The high death rate among women makes it the second deadliest malignancy after lung cancer [5, 6]. A study by the National Institute of Cancer in China found 1.67 million breast cancer and 522,000 deaths cases from 2008 to 2012 [7].

Despite extensive efforts from medical professionals and researchers, a definitive method for treating breast cancer has yet to be established and reliable evidence for its prevention remains elusive [8–11]. Some components of breast cancer tissues are highly malignant and pose a severe danger to patients' lives as they can spread to other vital organs [12–15]. The growth of mammary cells can lead to tumors in women. Tumors are classified as benign or malignant based on the area, size, and location, using the BI-RAD scores [16, 17]. Benign tumors are not life-threatening and can be treated through medication to prevent further growth [17, 18]. Malignant tumors, on the other hand, can spread to other parts of the body via the lymphatic system or blood, making them much more dangerous [19–22]. This uncontrolled cell proliferation in the breast leads to the formation of malignant tumors, which can only be treated through surgery or radiation therapy [23, 24].

Early detection of breast cancer is crucial for accurate diagnosis and analysis, and many researchers are turning to biomedical imaging to aid specialist radiologists. Various methods such as MRI, mammography, and ultrasound are utilized to identify breast carcinoma [25, 26]. However, the large volume of images challenges radiologists in identifying potential cancerous areas. Therefore, an efficient automated method is needed, and computer-aided diagnostic (CAD) systems are being utilized in aiding radiologists in detecting cancerous breast tumors [27, 28].

Increasingly, deep learning techniques are being applied to medical imaging to develop automated computer-aided diagnosis (CAD) systems [29–35]. Deep learning is considered the most effective method for detecting and classifying medical images [29, 30]. With these techniques, the mammogram image's significant low to high-level hierarchical features can be directly extracted, making deep learning the most reliable medical imaging method [29]. Several CAD systems based on deep learning have been developed for breast lesions detection, which outperforms traditional systems [36]. Accurate detection of breast lesions is crucial for improving breast cancer diagnosis [29, 31]. However, detecting these lesions can be challenging due to their varying texture, shape, position, and size. Deep learning and image processing methods have been proposed to overcome the limitations of conventional technology, which cannot perform automated identification [29]. The final stage in the CAD model is the classification of breast lesions into benign or malignant, which is important in assessing the correctness of the diagnostic [30].

Currently employed methods for detecting breast cancer are slow, costly, and require extra efforts to run the radiology equipment. Accurately detecting breast cancer automatically from an image processing perspective is not easy. Hence, early diagnosis and proper treatment are deemed crucial. Therefore, an efficient screening system and automation are necessary for breast cancer detection due to the following reasons [12]: incorrect diagnoses and predictions, tumors appearing in low contrast areas, unreliable human diagnoses, overburdening of radiologists, human error in diagnosis, need for large training data to avoid overfitting in deep learning algorithms, high computational complexity, and longer processing time for accurate tumor identification.

A novel breast cancer detecting system is proposed with an improved architecture that integrates deep convolutional neural network (DCNN) and breast mammogram images to address previous drawbacks mentioned above. The proposed system intends to divide breast tumors into benign and malignant categories. The system's performance is evaluated and compared with existing classification systems using a public mammographic image dataset named INbreast. The new system includes transfer learning to fine-tune the pretrained DCNN and detailed results from experiments on the INbreast dataset. The system's performance is evaluated using the following metrics: AUC, specificity, accuracy, sensitivity, and F-1 score.

The rest of the paper is organized as follows.  Section 2 presents the related work.  Section 3 provides the proposed approach for breast cancer detection.  Section 4 presents experimental analysis, results and comparison with existing work.  Section 5 concludes this paper and presents future work.

## 2. Related Work

Breast cancer diagnosis in modern medical procedures often involves using mammography images [37]. A summary of recently developed systems for breast cancer diagnosis using mammogram images is presented in this section.

Structured support vector machine (SSVM) and conditional random field (CRF) are two structured prediction techniques proposed in [38] to classify mass mammograms. Both approaches used potential functions based on deep convolution and belief networks. The results demonstrated that the CRF method outperformed the SSVM method in training and inference time. Authors in [29] utilized four-fold cross-validation on X-ray mammograms from the INbreast dataset to estimate a full-resolution convolutional network (FrCN). It resulted in an F1 score of 99.24%, an accuracy of 95.96%, and a Matthews correlation coefficient (MCC) of 98.96%. In another study [39], the BDR-CNN-GCN approach was proposed by combining a graph-convolutional network (GCN) with a basic 8-layer CNN that includes batch normalization and dropout layers. The final BDR-CNN-GCN model was formed by integrating the two-layer GCN with the CNN. This method was tested using the MIAS dataset, and successful results were obtained with a 96.10% accuracy level.

Authors in [40] proposed modifying the YOLOv5 network for identifying and classifying breast cancers, with the algorithm run using specific parameter values. The modified YOLOv5 was compared with a faster RCNN and YOLOv3, achieving an accuracy of 96.50% and an MCC value of 93.50%. The diverse features (DFeBCD) method was proposed by [41], which classified mammograms into two categories normal and abnormal. They used two classifiers, an emotion learning-inspired integrated classifier (ELiEC) and SVM, with the IRMA mammography dataset. The ELiEC classifier outperformed SVM, achieving an accuracy rate of 80.30%. In [30], a deep-CNN model that utilized transfer learning (TL) was introduced to prevent overfitting when working with small datasets. DDSM, MIAS, BCDR, and INbreast were used to assess its performance. INbreast dataset achieved an accuracy of 95.5%, the DDSM dataset achieved an accuracy level of 97.35%, and the BCDR database achieved a 96.67% accuracy level.

Authors in [42] for extracting features from breast mammograms utilized lifting wavelet transform (LWT). Feature vectors' size was reduced using linear discriminant analysis (LDA) and principal component analysis (PCA). The classification was performed using the moth flame optimization and extreme learning machine (ELM) approach with MIAS and DDSM and datasets, achieving accuracy of 95.80% and 98.76%, respectively. In addition, researchers have also trained the CNN Inception-v3 model on 316 images, resulting in a sensitivity of 0.88, specificity of 0.87, and an AUC of 0.946 [43]. Furthermore, in [44], a CNN and TL classification method was proposed to evaluate the performance of eight fine-tuned pretrained models. Authors in [45] presented a hybrid classification model using Mobilenet, ResNet50, and Alexnet with an accuracy level of 95.6%. In [46], four different CNN architectures (VGG19, InceptionV3, ResNet50, and VGG16) were utilized for model training using 5000 images, while prediction models were evaluated on 1007 images.

Authors in [47] utilized alpha, geostatistics, and diversity analyses forms in their proposed breast cancer detection method. They employed the SVM classifier on MIAS and DDSM databases, which resulted in a detection accuracy level of 96.30%. The SVM classifier and gray level co-occurrence matrix (GLCM) were employed by [48] for detecting breast cancer abnormalities in the MIAS data set. Their method achieved an accuracy of 93.88% and surpassed the performance of the k-nearest neighbour (kNN) algorithm. Authors in [49] used AlexNet and SVM to enhance classification accuracy with data augmentation techniques. The method achieved 71.01% accuracy, which increased to 87.2% with SVM and was evaluated on DDSM and CBIS-DDSM datasets.

A DenseNet deep learning framework extracted image features and classified cancerous and benign cells by feeding them into a fully connected (FC) layer. The effectiveness of this technique was evaluated by adjusting the hyperparameters [50]. An algorithm named DICNN was developed by Irfan et al. [51], which uses a dilated semantic segmentation network and morphological operation. Combining these feature vectors with SVM classification yielded an accuracy of 98.9%.

Although prior breast cancer detection and classification systems have improved information extraction, several issues still need attention, such as low contrast in tumor location, high memory complexity, long processing time, and the need for a large amount of training data for deep learning approaches. In response to these problems, we propose a new approach to breast cancer detection and classification, which will be discussed in detail in the following section.

## 3. Methodology

**Figure 1:**
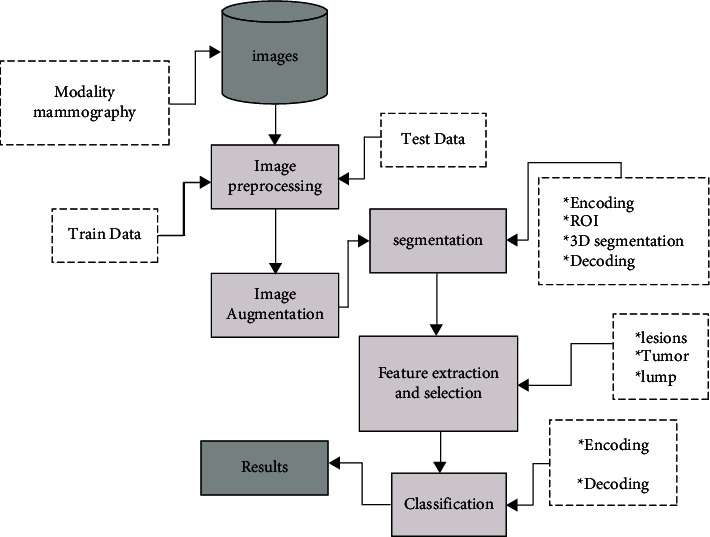
Architecture of the proposed system.

In this section, the processes used for implementing our proposed scheme are described in depth. The system consists of the following steps: (1) image enhancement, (2) image segmentation, (3) feature extraction and the selection, and (4) feature classification. The proposed system is illustrated in Figure 1.

### 3.1. Dataset

This study used a digital breast X-ray database named INbreast to implement the proposed CAD approach. The INbreast dataset is a public database that contains more recent FFDM images. It typically has an image size of 3328 × 4084 pixels. It contains 115 patients' cases along with 410 mammograms with both craniocaudal (CC) view and a mediolateral oblique (MLO) view. Of these 115 patients, 90 had mammograms taken of both breasts, totaling 360 images, while the other 25 had only two mammograms taken each. In total, 410 mammograms were produced from 115 patients, including cases of normal, benign, and malignant breasts. 107 cases with breast lesions were used from the MLO and CC views for evaluation purposes.

### 3.2. Convolutional Neural Network

**Figure 2:**
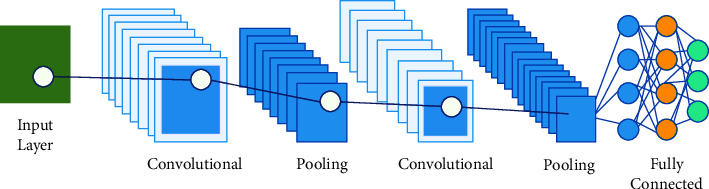
Standard architecture of CNN.

This subsection will examine the fundamental structure of all convolutional neural network (CNN) architectures. CNNs are deep neural networks used for image recognition and classification. In recent years, CNNs have become a crucial tool in image analysis, especially for identifying faces, text, and medical imaging. CNNs have a long history of success in image classification and segmentation, first developed in 1989. CNNs replicate the human brain's visual information processing by incorporating layers of “neurons” that only respond to their local surroundings. These networks can understand the topological aspects of an image through a combination of convolutional, pooling, and fully connected (FC) layers. The architecture of a CNN is shown in Figure 2.

#### 3.2.1. Convolutional Layers

The convolutional layers are assembled into feature maps based on local connections and weight distribution principles. A filter bank, a group of weights, connects neurons in a feature map to corresponding local regions in the preceding layer. Each feature map uses a different filter bank, and all the units in the map share the same filter row. This weight distribution and local connection help reduce the number of parameters by utilizing the close relationship between neighboring pixels and location-independent image features. The output of the weights is then sent to an activation function, such as ReLU or Sigmoid. This activation function enables the nonlinear transformation of the input data, which is necessary for the following processing stages.

#### 3.2.2. Pooling Layer

As illustrated in Figure 2, the pooling layer follows the convolution layer and uses subsampling to integrate the features from the convolutional layer into a single layer semantically. This layer's primary objective is to decrease the size of the image by combining pixels into one value while preserving its features. In this layer, typical operations include max as well as main pooling.

#### 3.2.3. Fully Connected Layer

The last layer in CNN is the dense classification layer, which is responsible for determining the category of input data based on extracted features from CNN. The number of units in the FC layer is the same as the number of different classifications (categories).

### 3.3. Proposed Workflow

This section provides the proposed workflow for breast cancer diagnosis using a deep convolutional neural network.

#### 3.3.1. Image Enhancement

**Figure 3:**
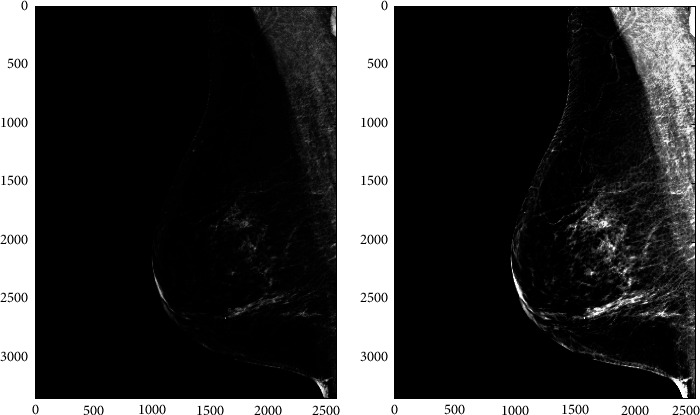
Contrast enhancement for improve visibility.

Image enhancement refers to increasing contrast and suppressing noise in mammogram images to assist radiologists in detecting breast abnormalities. Various image enhancement methods exist, including adaptive contrast enhancement (AHE). AHE improves the local contrast and reveals more image details, making it a helpful technique for enhancing both natural and medical images [52]. However, it may also result in considerable noise. In this paper, we utilized the contrast-limited adaptive histogram equalization (CLAHE) technique, a form of AHE, to enhance image contrast [52]. A drawback of AHE is that it can over-enhance the images due to the integration process [49]. To mitigate this issue, CLAHE is used as it limits the local histogram by setting a clip level, thus controlling contrast enhancement.  Figure 3 illustrates an image enhanced by the CLAHE algorithm.

Furthermore, CLAHE algorithm steps are given as follows [53]:

1. Split image into equal-sized contextual regions.
2. Apply histogram equalization to all contextual regions.
3. Limit the histogram to the level of the clip.
4. Reallocate the clipped values in the histogram.
5. Obtain enhanced pixel value through histogram integration.

### 3.4. Image Segmentation

Image segmentation involves dividing an image into regions with similar characteristics and features. Segmentation aims to simplify the image for easier analysis [54]. Popular image segmentation techniques include edge detection, partial differential equation (PDE), fuzzy theory, artificial neural network (ANN), region-based segmentation, and thresholding.

#### 3.4.1. Thresholding Method

One of the simplest image segmentation methods is the thresholding method [55, 56]. The pixels of the image are split according to their intensity level. The global threshold is the most commonly used thresholding technique [57]. It is accomplished by setting a threshold value (*T*) constant throughout the image. The output image is derived from the original image based on the threshold value.

#### 3.4.2. Region-Based Segmentation Methods

It is a simple approach compared to other methods, as it involves dividing an image into different sections based on predetermined. Compared to others, it is a straightforward method because it entails separating an image into different sections based on predetermined criteria [58]. There are two primary kinds of region-based segmentation: (1) region splitting and merging and (2) region growing. Region growing allows the removal of a region from an image using defined criteria, such as intensity. It involves selecting a starting seed point. It is important to note that unlike region growing, region splitting and merging work on the entire image [59].

**Figure 4:**
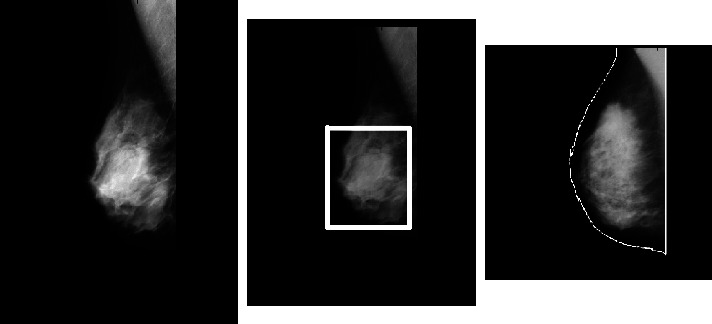
Defining region of interest (ROI).

In the present study extracting the region of interest (ROI) involves using both thresholding and region-based techniques. The tumor in the INbreast dataset samples cites moreira2012inbreast is labeled by a white bounding box, as shown in Figure 4. For extracting ROI, the tumor region is first determined by setting a threshold value based on the white color pixels in the image. The threshold for all images is determined to be 80 after several attempts, independent of tumor size. After identifying the greatest area inside this threshold within the image, the tumor is automatically cropped. Figure 4 shows ROI extracted using threshold and region-based methods.

The method for extracting ROI can be summarized in four steps:

1. Thresholding the grayscale mammogram image to create a binary image.
2. Labelling and counting the binary image objects, then retaining only the largest one, which is the tumor, as defined by the white bounding box.
3. Assign the largest area within the threshold value to “1” and the rest a value of “0.”
4. Multiply binary image with original mammogram image for obtaining final ROI without including other parts of breast or artifacts.

#### 3.4.3. Feature Extraction and Selection

Numerous methods exist for feature extraction. Due to their exceptional performance, deep convolutional neural networks (DCNN) garnered significant interest in recent years. Consequently, the DCNN is utilized in this paper.

#### 3.4.4. Deep Convolutional Neural Network

The success of DCNN in image classification and analysis has been documented in various studies [60, 61]. Convolutional neural networks (CNNs) are composed of multiple trainable stages that culminate in a supervised classifier and feature maps [62]. Three primary types of layers are employed to build CNN structures: convolutional, pooling, and fully connected (FC) layers [63]. The ResNet50 CNN classification model categorizes breast cancer as benign or malignant in this work.

#### 3.4.5. Feature Learning through Transfer Learning

Machine learning has various feature learning methods (FL), allowing a system to automatically identify the representations required for feature detection, prediction, or classification from a preprocessed dataset [64]. This implies that the machine can learn and use the features to perform tasks such as classification or prediction. In deep learning, FL can be accomplished by constructing a complete CNN to train and test image datasets or adjust a pretrained CNN for classification or prediction on a new image dataset, referred to as transfer learning.

In deep learning, transfer learning (TL) is a widely-used technique that enables the utilization of a pretrained network for new prediction or classification tasks. This is achieved by adjusting the parameters of the pretrained network with randomly initialized weights for the new task. TL typically results in faster training than starting from scratch and is considered an optimization that saves time and improves performance, as stated in [65]. For this purpose, transfer learning is utilized to fine-tune ResNet50 CNN. This involves using pretrained weights from the ImageNet dataset [66] for retraining after preprocessing the collected dataset. The network parameters and hyperparameters are optimized during this process.

#### 3.4.6. Classification

The features are taken from ResNet-50 and processed via a fully connected (FC) layer with a 40% dropout rate to avoid overfitting [67, 68]. This layer is then activated with the rectifying function, ReLU. All negative values are set to zero in the input matrix, while other remains unchanged. The use of ReLU leads to faster and more reliable convergence than a sigmoid activation function during training deep networks [69]. The output layer comprises a sigmoid function (binary classifier) to provide class probabilities. The sigmoid function normalizes the input into two outcomes, i.e., malignant vs. benign [70].

## 4. Evaluation and Results

The proposed deep convolutional neural network for mammogram imaging undergoes examination and validation in this section. Information about benchmark datasets, assessment metrics, and comparisons to other leading techniques are also covered.

### 4.1. Image Acquisition Process

The proposed system's performance is evaluated using digitized mammogram images from the INbreast dataset [71]. The database is used to demonstrate the efficiency and reliability of the proposed method for identifying breast cancer. INbreast dataset includes 336 mammogram images, with 269 abnormal and 69 normal images, where 220 are benign and 49 malignant cases. Tables 1 and 2 show the distribution of mammography images.

### 4.2. Metrics of Performance

The purpose of cross-validation is to improve efficiency, validate performance, and assess the results from the dataset. To assess the classification efficiency of the proposed method, multiple metrics are utilized such as confusion matrix, accuracy, sensitivity, specificity, error rate, F1 score, and area under the curve (AUC). All these metrics act as benchmark values for comparing the proposed method against previous algorithms [72]. These measurements are defined as follows.

#### 4.2.1. Confusion Matrix

Confusion matrix represents the performance of a classifier in the form of a table. In ML, it is also known as an error matrix. The image regions are labeled positive or negative based on the data type. The classifier's decision can be correct (true) or incorrect (false). This results in four outcomes: true positive (TP), true negative (TN), false positive (FP), and false negative (FN). Correct decisions are represented along the diagonal of the confusion matrix.

#### 4.2.2. Accuracy

Accuracy characterizes the suitable labeled images for benign, normal, and malignant mammograms. The accuracy of the process is computed as follows:

(1) $$\begin{array}{c} \text{Accuracy}=\frac{\text{TP}+\text{TN}}{\text{TP}+\text{FP}+\text{TN}+\text{FN}}\text{.} \end{array}$$

TP accurately represents positive examples; TN addresses classified negative examples; TN means incorrectly classified examples as accurately classified; and FN indicates accurately classified examples as the wrong sample.

#### 4.2.3. Specificity

The chances that the test will correctly recognize the patient who has the disease is shown in the following equation:

(2) $$\begin{array}{c} \text{Specificity}=\frac{\text{TN}}{\text{TN}+\text{FP}}\text{.} \end{array}$$

#### 4.2.4. Sensitivity

The chance that the test will correctly recognize a patient with the disease is shown in equation:

(3) $$\begin{array}{c} \text{Sensitivity}=\frac{\text{TP}}{\text{TP}+\text{FN}}\text{.} \end{array}$$

#### 4.2.5. F1 Score

It is a weighted average of precision and recall used for assessing the classifier's performance. It considers both false positives and negatives in its calculation, as shown in the following equation:

(4) $$\begin{array}{c} F1\text{score}=2*\frac{\text{Precision}*\text{Recall}}{\text{Precision}+\text{Recall}}\text{.} \end{array}$$

#### 4.2.6. Area Under the Curve (AUC)

AUC is the classifier's ability to distinguish between benign, normal, and malignant mammograms.

## 5. Results and Discussion

For this study, a subset is taken from the INbreast dataset, and each sample is increased to four images. During the experiment, 60% images were used for training, and the remaining 40% were used for testing. The samples were first subjected to enhancement and segmentation according to the procedures described in the “Methodology” section. Afterward, features were extracted from the samples using a CNN. Finally, all the samples were classified using ResNet-50.

The proposed DCNN method categorizes mammogram images of breast tumors into benign or malignant. A dataset named INbreast is used for experimentation.  Table 3 displays the classification accuracy achieved by the proposed ResNet-50 method across the INbreast database. From the INbreast dataset, 132 benign and 29 malignant image samples were selected for training, and 20 malignant and 88 benign for testing. The resulting accuracy is 93%. The proposed ResNet-50 approach is also compared quantitatively with previously existing algorithms. The study's results revealed that the presented approach outperformed these algorithms with high accuracy, specificity, F1 score values, and sensitivity.

As shown in Table 3, the proposed approach demonstrated improved results on the INbreast database with an accuracy of 93.0%, specificity of 93.86%, and sensitivity of 93.83%. It outperforms other methods in terms of accuracy. Although the accuracy achieved by [16] is slightly higher at 91.0%, the proposed approach still exhibits the best performance compared to the other methods. Compared to existing methods, the proposed approach enhances breast cancer detection and classification performance. It can potentially be used for real-time evaluation and to support radiologists in automating the analysis of mammogram images. However, performance may vary when the same method is applied to different datasets due to factors such as background noise, lighting conditions, occlusion, overfitting, and the nature of the method.

**Figure 5:**
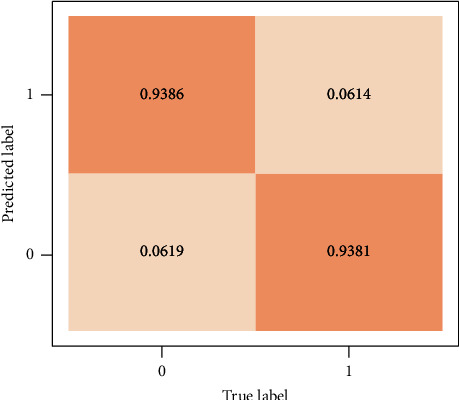
Confusion matrix of the proposed approach for classification on INbreast database.

**Figure 6:**
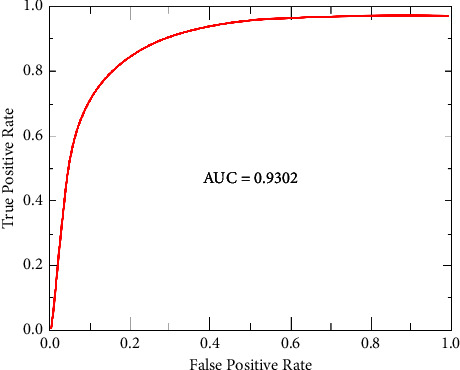
ROC plot on INbreast dataset.

The performance of the presented approach is also evaluated using the confusion matrix and ROC curves.  Figure 5 illustrates the confusion matrix on the INbreast data set. AUC, a crucial statistical metric in the ROC curve, is computed using the INbreast data set. Metric in the ROC curve is calculated INbreast data set. ROC curves were constructed based on true positive rate (sensitivity) and false positive rate (1-specificity) rates, controlled by the threshold of the obtained probability maps.  Figure 6 shows the ROC curve graph.

Table 4 presents our proposed system's results of breast cancer detection. The proposed approach achieved an F1 score and AUC of 93.03% and 93.02%, respectively, on the INbreast database.

In recent years, breast cancer detection and classification applications have gained widespread use in the medical field, making the diagnostic process more accurate [76, 77]. The goal of the proposed method is to enhance clinical diagnosis by enhancing the detection of breast cancer. The opinions of two medical specialists were gathered based on the accuracy level generated by our proposed algorithm. These experts expressed their appreciation for the improved results of ResNet-50 compared to other approaches. To sum it up, the proposed approach enhances performance compared to other methods and can be utilized for real-time evaluations along with helping radiologists automate the evaluation of mammograms.

## 6. Conclusion

The proposed system aimed to detect malignant breast masses and classify benign and malignant tissues in mammograms. A novel computer-aided detection (CAD) system is proposed, which involves thresholding and region-based segmentation techniques. A region-based method with a threshold of 80 determines the largest area included in this threshold. A deep convolutional neural network (DCNN) is utilized during feature extraction. Specifically, the ResNet-50 is retrained to classify the mammograms into two classes (malignant or benign), and its parameters were modified to classify breast mammograms. The proposed approach is applied to the INbreast database to evaluate its performance of the proposed approach. The proposed method achieved an accuracy of 93.0%, specificity of 93.86%, AUC of 93.02%, a sensitivity of 93.83%, and an F1 score of 93.03%, which are extremely satisfying results. The proposed method surpasses the detection and classification of mammograms, delivering more precise results and improved visual outcomes compared to other systems. The proposed system efficiently detects and classifies malignant breast masses with reduced computation time and produced satisfactory results. Alternative networks, such as deep convolutional networks (VGG) and AlexNet architecture, will be proposed for future development. In the future, we intend to extend this work by collecting large datasets on breast cancer in different age intervals to detect cancer in its early stages.

## Figures and Tables

**Table 1:** Normal and abnormal INbreast dataset.

Total images (normal)	Total images (abnormal)	Training (normal)	Training (abnormal)	Testing (normal)	Testing (abnormal)
67	269	40	162	27	107

**Table 2:** Benign and malignant INbreast dataset.

Total images (benign)	Total images (malignant)	Training (benign)	Training (malignant)	Testing (benign)	Testing (malignant)
220	49	132	29	88	20

**Table 3:** Summary of breast cancer detection on different mammography datasets.

Authors	Dataset	Method	Accuracy (%)	Specificity (%)	Sensitivity (%)
Carneiro et al. [73]	INbreast	AlexNet	86	N.A.	N.A.
Dhungel et al. [16]	INbreast	AlexNet	91.0	N.A.	N.A.
Zhang et al. [74]	INbreast	AdaBoost	87.93	97.73	57.20
Pezeshki et al. [75]	MIAS	ANN	61.0	77.0	95.03
Zhang et al. [74]	DDSM	AdaBoost	90.91	97.38	82.96
Proposed approach	INbreast	ResNet-50	93.0	93.86	93.83

**Table 4:** Results of our proposed methodology for breast cancer detection and classification.

Performance metrics	Result obtained (%)
Accuracy	93.0
Sensitivity	93.83
Specificity	93.86
F1 score	93.03
AUC	93.02

## Data Availability

The (Breast Cancer Diagnosis) data used to support the findings of this study are included within the article.
